# Cryogenic Foaming of Silk Fibroin Composite for Scaffolds in Bone and Periodontal Regeneration

**DOI:** 10.3390/jfb17050230

**Published:** 2026-05-06

**Authors:** Giuseppe De Giorgio, Barbara Medagli, Biagio Matera, Katia Rupel, Giuseppe Tarabella, Gianluca Turco, Maddalena Manfredi, Benedetta Ghezzi, Pasquale D’Angelo

**Affiliations:** 1 Institute of Materials for Electronics and Magnetism, CNR, Parco Area delle Scienze 37A, 43124 Parma, Italy; giuseppedegiorgio@cnr.it (G.D.G.); giuseppe.tarabella@cnr.it (G.T.); pasquale.dangelo@cnr.it (P.D.); 2 Clinical Department of Medical, Surgical and Health Sciences, University of Trieste, 34100 Trieste, Italy; bmedagli@units.it (B.M.); krupel@units.it (K.R.); gturco@units.it (G.T.); 3 Centre of Dental Medicine, Department of Medicine and Surgery, University of Parma, Via Gramsci 14/A, 43126 Parma, Italy; maddalena.manfredi@unipr.it

**Keywords:** silk fibroin, PCL, bone regeneration, β-TCP, scaffold, cryogenic mechanical foaming

## Abstract

Bone tissue has a remarkable regenerative capacity; however, advanced strategies are needed to support the repair process for critical-sized defects. While autografts and allografts remain the gold standard, their limitations have stimulated alternative approaches in bone tissue engineering, in search of scaffolds capable of mimicking native bone properties to promote effective regeneration. In this study, silk fibroin (SF)-based composite scaffolds incorporating β-tricalcium phosphate (β-TCP) and poly-ε-caprolactone (PCL) were synthesized using a simple and innovative cryogenic foaming method. The proposed fabrication technique overcomes many limitations of current synthesis methods, such as long processing times, the use of solvents, and reliance on complex, energy-intensive equipment. The composites were characterized using infrared spectroscopy to confirm the incorporation of all three components and their chemical bond arrangements. µ-CT, SEM, and ESEM analyses revealed that SF/β-TCP/PCL scaffolds exhibited great porosity and dynamic interaction with water while preserving pore morphology in wet environments. Swelling behavior, indirect cytotoxicity, and cell proliferation tests recognized the greater performance of SF/β-TCP/PCL scaffolds in promoting long-term cell proliferation, maintaining superior mechanical properties. These findings indicate that the proposed original, simple, and relatively low-cost manufacturing approach enabled the fabrication of scaffolds with excellent mechanical performances, controlled and stable porosity under both dry and physiological-like conditions, and high biocompatibility. The resulting constructs demonstrated promising results for cell proliferation and osteoconductive behavior, supporting their potential suitability as artificial bone substitutes.

## 1. Introduction

Bone tissue has a well-recognized ability to regenerate. Nevertheless, advanced clinical strategies are needed to promote repair in challenging conditions, particularly in critical-sized defects that hinder natural bone formation.

Nowadays, autografts and allografts remain the gold standard for treating bone defects, as they support regeneration by enabling direct bone bonding (i.e., osteoconduction) while simultaneously inducing local stem cells to differentiate into bone-forming cells (i.e., osteoinduction), all while minimizing immune reactions [1,2]. However, significant limitations of this therapeutic approach may arise from the limited availability of suitable donor tissue, the need of a second surgical procedure to harvest the graft, and the risk of severe complications associated with the use of non-autologous tissues [3,4].

These drawbacks have driven the development of alternative strategies that employ synthetic or natural biomaterials designed to promote bone regeneration while overcoming the inherent limitations of graft-based therapies.

In this light, artificial bone scaffolds must meet specific requirements, such as (i) excellent biocompatibility, (ii) a porous and interconnected structure, along with (iii) adequate mechanical properties. Each of these factors plays a pivotal role in determining the overall success of the synthetic graft. For instance, while an interconnected porous structure can promote cell infiltration and tissue regeneration, it may simultaneously compromise the scaffold’s overall mechanical strength, thus affecting its degradation rate.To develop truly representative bone tissue scaffolds, it is essential to mimic the hierarchical organization of native bone, together with the right balance of organic–inorganic composition [5].

Therefore, the choice of biomaterials is crucial. Bone tissue engineering (BTE) offers a wide range of options, which can be broadly categorized into natural and synthetic polymers. Natural biopolymers are characterized by enhanced biocompatibility, improved support for cellular functions, non-cytotoxicity, and biodegradability compared to the synthetic ones [6]. To mimic the organic phase of the bone, silk fibroin (SF) from Bombyx mori cocoon and poly-ε-caprolactone (PCL) have been widely used for in vitro and in vivo applications due to their tailorable bioactivity and mechanical properties, being also able to guarantee a gradual replacement with newly formed tissue [7,8,9]. However, currently available SF and PCL scaffolds exhibit limited osteoconductive and osteoinductive properties and a tendency toward surface hydrophobicity, which can hinder the adsorption of extracellular matrix (ECM) proteins, thus leading to decreased osteoblasts adhesion rate [8].

Combining with calcium-based materials is required to overcome some of these limitations, improving the mechanical strength of the structure and enabling the controlled release of calcium ions during scaffold degradation [6].

Although it is well established that the inorganic phase of the bone is mainly composed of hydroxyapatite (HA), β-tricalcium phosphate (β-TCP) has emerged as a promising alternative due to its higher resorbability [10,11]. Various methods have been explored to combine these materials and produce porous structures, such as gas foaming [12,13] salt-leaching [14], electrospinning [15], freeze-drying [6], and 3D printing [8,16]. All these techniques can provide one or more of the required properties; however, the fabrication of an ideal 3D scaffold has not yet been achieved. In particular, commonly employed methods such as freeze-drying, gas foaming, and salt-leaching suffer from several limitations, including insufficient control over pore size, shape, and overall porosity in the resulting scaffolds, as well as poor suitability for the fabrication of complex geometries [17,18].

In the current literature, mechanical foaming of SF is rarely explored, and there are no studies investigating this fabrication method for bone tissue engineering applications. For these reasons, in this work, we explored a variation in conventional mechanical foaming by incorporating a cryogenic step of pretreatment/flash-freezing in liquid nitrogen to minimize bubble collapse while maintaining a porous structured matrix. The use of liquid nitrogen as a cryogenic agent in scaffold fabrication has been relatively underexplored, despite its potential to enhance mechanical properties and to preserve scaffold architecture by promoting the formation of smaller ice crystals [19,20]. Our approach may help overcome several current limitations of SF-based composites for bone regeneration: (i) improved control of the overall porosity and pore morphology; (ii) the incorporation of high amounts of ceramic components; (iii) enhanced geometric versatility for the fabrication of complex structures, including the potential for 3D printability; (iv) the achievement of a high surface-to-volume ratio while maintaining mechanical properties under different conditions; and (v) the simplicity/quickness and cost-effectiveness of the method. The conceptual basis of the synthesis employed herein has been established in previous work [21]. In the present study, appropriate adjustments were introduced to obtain scaffolds with enhanced mechanical performances in wet environments and high β-TCP loading, biocompatibility and biodegradability, sustained cellular proliferation, and osteoconductivity. The proposed synthesis strategy allowed to produce SF-based scaffolds enriched with high β-TCP content and reinforced with a PCL core, acting as a structural stabilizer. The resulting synthetic grafts exhibited interconnected porosity and mechanical properties that remain stable in wet conditions. These characteristics, supported by preliminary in vitro biological outcomes, suggest the potential applicability of the proposed scaffold as artificial bone substitutes.

## 2. Materials and Methods

### 2.1. SF Preparation

Bombyx mori cocoons were boiled in a 0.02 M Na_2_CO_3_ solution (VWR, West Chester, PA, USA) for 20 min and thoroughly rinsed with Milli-Q water to completely remove sericin and residual wax, yielding degummed SF fibers. The fibers were dried overnight and dissolved in 9.3 M lithium bromide (LiBr; Alfa Aesar, Ward Hill, MA, USA) at 60 °C until complete dissolution. The solution was then dialyzed for 3 days against ultrapure water using dialysis membranes with a molecular weight cutoff of 10 kDa (Spectra/Por 6, VWR, Waltham, MA, USA). After dialysis, the aqueous SF solution was centrifuged at 4500 rpm for 20 min to remove impurities and undissolved residues. The SF solution was then concentrated by gentle stirring at 60 °C on a hot stir plate until reaching a 10 wt% working concentration, confirmed by drying a sample of the solution and weighing the residual solid.

### 2.2. Fabrication of SF-Based Cryogenic Composite Foams

Three types of SF-based composite foams were prepared, all using a 10 wt% SF solution as the base material: the CNT samples were prepared using only the SF solution, the SF/β-TCP samples were prepared using the SF solution with the addition of 30% *w*/*v* β-tricalcium phosphate (β-TCP; Merck, Darmstadt, Germany), and the SF/β-TCP/PCL samples were prepared using the SF solution with the addition of 30% *w*/*v* β-TCP and 10% *w*/*v* polycaprolactone (PCL; Evonik Industries, Essen, Germany). Each sample was vigorously whipped (1 min at 11,000 rpm) using a milk frother, and for the SF/β-TCP and SF/β-TCP/PCL samples, the powders were added to the SF solution prior to the foaming step, allowing complete incorporation of the components during whipping. Whipped foams were poured into plastic molds, and they were flash-frozen in liquid nitrogen. The flash-freezing step was followed by annealing at −20 °C overnight. The annealed sponges were then demolded and dried at 60 °C for 4 h.

Dried cryogenic foams were immersed in methanol for 1 h, inducing crystallization without macroscopic changes. This treatment is essential for triggering the conformational switch of SF to the silk II structure, promoting complete β-sheet crystallization and the transition from α-helices to β-sheets, which enhances the material’s mechanical properties [9]. Additionally, the SF/β-TCP/PCL samples were dipped in chloroform for 15 min to dissolve PCL. This treatment does not macroscopically alter the matrix patterns but causes PCL melting and redistribution within the scaffold pores, forming a PCL network. During the entire work, no significant macroscopic or physico-chemical variations were detected between different batch preparations, indicating the high reproducibility of the method.

### 2.3. FTIR-ATR Analysis

Dry samples were analyzed via Fourier-transform infrared spectroscopy (FTIR) in attenuated total reflection (ATR) mode using a Cary 630 spectrometer (Agilent, Santa Clara, CA, USA). The spectra were recorded in the 4000–700 cm^−1^ range. OriginPro 2018 software (OriginLab, Northampton, MA, USA) was used for the analysis and graphing of the full spectra, as well as for amide I and II bands.

### 2.4. Morphological Characterization

Scaffold morphology was assessed under both dry and wet conditions to investigate structural variations between the two states. Prior to the analysis, the samples were immersed in a Human Plasma-Like Medium (HPLM) for 24 h. The images of dry and wet specimens were acquired using a stereomicroscope (SMZ25, Nikon, Tokyo, Japan) for macroscopical assessment. For more detailed morphological and structural characterization, the analysis was conducted using a Zeiss Auriga compact field emission SEM (Carl Zeiss, Oberkochen, Germany) equipped with a GEMINI field-emission SEM column operated at 5 keV and a Quanta250 SEM (FEI, Hillsboro, OR, USA) operating in environmental mode. The working distance was adjusted to optimize magnification, with the acceleration voltage set at 30 kV.

The bulk density of scaffolds was determined through liquid immersion displacement, using ultrapure water as medium. To prevent liquid infiltration into pores, scaffolds were coated with Parylene C using a Parylene P6 coating system (Diener electronic GmbH, Ebhausen, Germany). Dry scaffolds were weighed ($m_{s}$), they were immersed in a graduated cylinder containing a known water volume ($V_{1}$), and the displaced volume was recorded as $V_{s}=V_{2}-V_{1}$, yielding apparent density:

$$\rho_{app}=\frac{m_{s}}{V_{s}}$$

This envelope volume method accounts for irregular geometries and sealed pore accessibility.

### 2.5. X-Ray Microcomputed Tomography (µ-CT)

µ-CT was performed using the custom cone-beam TOMOLAB system (Elettra Sincrotrone, Trieste, Italy [22]). The system is equipped with a sealed microfocus X-ray tube operating within an energy range of 40–130 kVp. A focal spot size of approximately 5 µm can be achieved when the source is operated at powers up to 4 W. In the present study, scans were acquired at 40 kV and 100 µA, corresponding to a source power of 4 W.

The images were collected using an sCMOS detector coupled to a fiber optic taper, with a native pixel size of 13.2 µm and a 4096 × 4096 pixel matrix. The detector was operated in 2 × 2 binning mode, resulting in an effective detector pixel size of 26.4 µm. With the adopted acquisition geometry, consisting of a source-to-object distance (FOD) of 90 mm and a source-to-detector distance (FDD) of 264 mm, this configuration yielded an equivalent voxel size of approximately 9 µm.

The samples, either dried or after 24 h of incubation in PBS at 37 °C, were mounted on the rotation stage, and they were scanned under the following conditions: FOD, 90 mm; FDD, 264 mm; binning, 2 × 2; equivalent voxel size, 9 µm; projection image size, 1984 × 1984 pixels; reconstructed slice size, 1984 × 1984 pixels; number of projections used for reconstruction, 1801; tube voltage, 40 kV; tube current, 100 µA; and exposure time, 1 s per projection. Projection images were cropped from the detector active area before reconstruction. The acquired images were normalized to the detector’s maximum intensity after the subtraction of the dark field.

Tomographic reconstruction was performed using CERA reconstruction software version 7.0, with X-Explorer as the graphical user interface (Siemens Healthineers, Erlangen, Germany). Both projection data and reconstructed slices were stored as 16-bit integer images, with one file per projection and one per reconstructed slice.

Image processing and segmentation were performed in FIJI/ImageJ version 1.54r using the BoneJ plugin [23]. Threshold-based segmentation was carried out according to Otsu’s method [24] to quantify structural parameters, including porosity, interconnectivity, pore size, and trabecular thickness [25,26]. For each sample, three VOIs with dimensions 300 × 300 × 300 pixels were considered.

### 2.6. Swelling/Weight Stability Experiments

Swelling ratio (Sr) was measured gravimetrically. Dry samples were weighed (W_d_), then immersed in ultrapure water at 37 °C for 24 h, after which excess water was gently removed through capillarity using filter paper, and the samples were weighed to obtain their wet weight (W_w_). The S_r_ (%) for each sample was calculated as follows:

$$S_{r}=\frac{W_{w}-W_{d}}{W_{d}}\times 100\%.$$

Total retained water (R_w_) was measured gravimetrically by weighing samples swelled for 24 h at 37 °C in ultrapure water, without removing excess water, to obtain W_1_. W_1_ was compared to the weight after removing excess water through capillarity (W_2_). R_w_ (%) was calculated for each sample according to the following equation:

$$R_{w}=\frac{W_{2}-W_{1}}{W_{1}}\times 100\%.$$

This parameter provides information on the total amount of water interacting with the scaffold’s surfaces and cavities, also reflecting the porosity maintenance after 24 h in a wet environment.

### 2.7. Weight Stability Test

Weight stability was assessed by incubating scaffolds in sterilized PBS (0.01 M; Merck, Darmstadt, Germany) at 37 °C. Wet weights of the scaffolds were measured at different time points after the removal of excess water through capillarity using filter paper, and the percentage gain/loss in weight for each condition was calculated relative to the measured day 1 wet weight.

### 2.8. Uniaxial Compression Tests of Scaffolds

A universal Testing Machine (Galdabini Sun 500, Cardano al Campo, VA, Italy) coupled with a 100 N load cell was used to test the produced scaffolds, both dry and after 24 h in PBS at 37 °C. A 1 mm/min deformation rate was applied through a cylindrical pressor (radius equal to 1 mm) on the upper surface of the samples. Load–displacement data were recorded during the test, and the stress/strain graph was plotted normalizing the recorded data on the surface of the pressor and the initial length of the scaffold. Compressive modulus was calculated in the 1–5% strain range (within the linear behavior of the material) of the stress–strain curves.

### 2.9. In Vitro Biological Testing

#### 2.9.1. Sample Preparation, Sterilization, and Equilibration

Prior to cell seeding, the obtained scaffolds were prepared by trimming their extremities with a razor blade to remove the SF skin and expose the lateral porosity. Moreover, polylactic acid (PLA) rings were 3D-printed and positioned on the scaffold to prevent scaffold flotation during the cultural period. Both scaffolds and 3D-printed PLA rings were sterilized using a multi-step protocol. First, all materials were exposed to ultraviolet (UV) irradiation for 30 min, with the process repeated three times. Subsequently, the materials were transferred to a 24-well plate and soaked in a 70% ethanol solution for 10 min, followed by various baths in a sterilization solution containing decreasing concentrations of amphotericin and penicillin/streptomycin (from 5% to 1% *v*/*v*) to avoid potential microbial contaminants. The sterilized scaffolds were equilibrated by the immersion in 1.5 mL of DMEM High Glucose media (Dulbecco’s Modified Eagle Medium; EuroClone, Pero, MI, Italy) supplemented with 2 mM l-glutamine (Thermo Fisher Scientific, Waltham, MA, USA), 10% *v*/*v* FBS (Fetal Bovine Serum, Thermo Fisher Scientific), and 1% *v*/*v* penicillin/streptomycin solution (Thermo Fisher Scientific) for 24 h prior to cell seeding. For longer constructs, the same procedure was applied, ensuring complete immersion of the scaffold in the culture medium to allow through diffusion and uniform equilibration throughout the entire structure.

#### 2.9.2. Cell Culture and Indirect Cytotoxicity Assay

Before evaluating the osteoblasts–scaffold interaction, an indirect cytotoxicity test was conducted to exclude the release of any cytotoxic agent from the graft, following the ISO 10933-5 (International Organization for Standardization, Geneva, Switzerland; ISO 10993-5:2018 Biological evaluation of medical devices—Part 5: Tests for in vitro cytotoxicity.) guidelines for the cytotoxicity analysis of porous materials. To this purpose, the scaffolds were immersed in RPMI 1640 (Thermo Fisher Scientific) supplemented with 10% *v*/*v* FBS and 1% *v*/*v* penicillin/streptomycin solution, under sterile conditions and incubated at 37 °C and 5% CO_2_ in a humidified atmosphere for 1, 7, and 10 days. At each experimental time point, the conditioned medium was collected from the scaffolds and diluted with pristine medium to reach the final concentration of 50%, 70%, and 100%. Conditioned medium was then added to previously seeded murine fibroblasts (L929; 5.000 cells/100 µL). The cells were then cultured for an additional 24 h, at the end of which the metabolic analysis through MTT test was performed.

In parallel, the biological analysis aimed to investigate cell–scaffold interactions, which were performed using the human MG-63 osteoblast cell-line (ATCC, Manassas, VA, USA, distributed by LGC Standards srl, Milan, Italy; number: CRL1427). The cells were cultured in DMEM High Glucose, supplemented with 2 mM l-glutamine, 10% *v*/*v* FBS, 100 U penicillin, 0.1 mg/mL streptomycin and maintained into 25 cm^2^ culture flask at 37 °C in a humidified 5% CO_2_ atmosphere.

#### 2.9.3. Osteoblasts Seeding on Scaffolds for Adhesion and Proliferation Assays

Before cell seeding, the medium was removed from each well, and the scaffold was gently blotted on a sterile gauze to remove excess liquid. MG-63 cells were then seeded onto the top of each scaffold at a density of 1 × 10^5^ cells per sample, in a 50 µL volume of complete DMEM High Glucose medium. After an initial incubation period of 4 h at 37 °C in a humidified 5% CO_2_ atmosphere, 1.5 mL of fresh culture medium was carefully added to each well. The culture medium was subsequently replaced every 3 days. Cell adhesion and proliferation were assessed through an MTT assay after 24 h for the first test and 24 h and 7 days for the second one.

#### 2.9.4. MTT Assay

Tetrazolium salts test (MTT) (Sigma Aldrich, Burlington, MA, USA) was used to evaluate cell metabolic activity. At each time point (1 and 7 days), the scaffold was moved to a new well plate, a solution of 0.5 mg/mL of MTT in DMEM High Glucose (1 mL/scaffold) was added, and it was incubated for 4 h at 37 °C in 5% CO_2_ in the dark. Purple formazan was eluted in 1 mL dimethyl-sulfoxide (DMSO) for 30 min at room temperature. A total of 200 µL were transferred to a 96-well transparent plate and absorbance (OD = 560 nm and 630 nm) was measured using a spectrophotometer (Varioskan LUX multimode microplate reader, Thermo Scientific, USA). The absorbance of the formazan product formed is directly proportional to the living cells on the porous scaffold. Cell-free scaffolds were used as blanks.

#### 2.9.5. Stereoscope and Confocal Microscope Images

Cell viability was assessed using the MTT assay (as described). The images were acquired using a stereomicroscope (Leica MZ16, Leica Microsystems, Wetzlar, Germany) to evaluate cell colonization. The presence of viable cell clusters was indicated by the formation of purple formazan crystals. Image processing and analysis were performed using Image-Pro Plus 6.2 software (Media Cybernetics, Rockville Pike, MD, USA).

For confocal imaging, the cells on the scaffold surface were fixed with 4% PFA in PBS for 20 min and washed twice with PBS. ActinGreen 488 (Life Technologies, Thermo Scientific) was used to label the actin filaments according to the manufacturer protocol. Following 2 washes in PBS, the images were collected using a spectral confocal laser microscope (Eclipse C1si, Nikon).

### 2.10. Statistical Analysis

The data were analyzed using Prism9 (GraphPad, La Jolla, CA, USA) and were reported as the average ± SEM (standard error of the mean). The differences between groups were evaluated with ordinary one- and two-way ANOVA statistical tests, followed by a Tukey post hoc test for multiple comparisons. The differences were considered significant when *p* < 0.05. All experiments were performed in triplicate.

A *post hoc* power analysis was performed using G*Power 3.1 to estimate the achieved statistical power of the performed one- and two-way ANOVAs. The analysis was conducted using an effect size derived from the pilot data, a significance level of α = 0.05, and a desired power of 0.80. For the one-way ANOVA, the F-test family was selected with the statistical test defined as “ANOVA: fixed effects, omnibus, one-way”. For the two-way ANOVA, the F-test framework was applied, with separate analyses performed for each main effect and their interactions. Effect sizes were calculated from the observed ANOVA results and expressed as Cohen’s *f* for each effect. The analysis indicated a high statistical power across all tests, with values ranging from 0.85 to 1, supporting the robustness and reliability of the detected effects.

## 3. Results and Discussion

### 3.1. Synthesis and Characterization of Silk Fibroin Cryogenic Composite Foams

**Figure 1:**
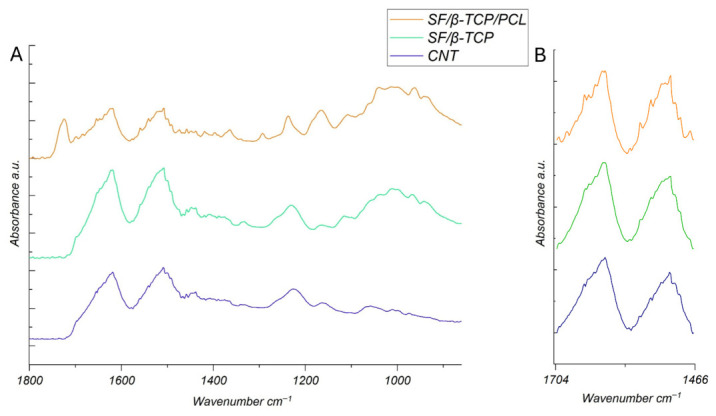
(**A**) ATR-FTIR spectra of the analyzed samples. CNT represents a control sample made up of only SF (purple curve), while SF/β-TCP (green curve) and SF/β-TCP/PCL (orange curve) represent the SF composites. (**B**) Normalized amide I and II peak spectral magnification, showing the persistence of a high degree of SF crystallization in all samples.

The successful completion of the synthesis and incorporation process, as described in Section 2, was validated through Attenuated Total Reflectance-Fourier Transform Infrared (ATR-FTIR) spectroscopy to (i) identify the constituent elements of the composite scaffolds, as well as to (ii) analyze the arrangement of their chemical bonds and (iii) secondary structure. The overlaid FTIR spectra of the three samples are reported in Figure 1A.

The characteristic SF vibrational modes, such as amide I (around 1650 cm^−1^), amide II (around 1530 cm^−1^), and other distinct bands in the fingerprint region (below 1500 cm^−1^) are clearly present in all samples. In the SF/β-TCP spectra, β-TCP incorporation is confirmed by the appearance of new peaks or shoulders, especially in the phosphate region between approximately 1000–1100 cm^−1^, which is characteristic of inorganic phosphate groups in the FTIR analysis [27]. Upon PCL incorporation, further spectral changes are observed, including new bands or intensified shoulders, especially near 1720 cm^−1^ in the ester carbonyl region and in the aliphatic C–H stretching regions, reflecting the PCL molecular structure [28].

The FTIR spectra provides further insights into the conformational state of SF through the analysis of the amide I region (Figure 1B), which is particularly sensitive to secondary structure and β-sheet content. In all samples, the sharpness of the amide I and II peaks suggest a high β-sheet content induced by the methanol treatment, which promotes an extensive protein crystallization in the CNT samples [29].

A comparable spectral profile is observed after the incorporation of β-TCP and the combined addition of β-TCP and PCL, suggesting that β-sheet crystallinity is effectively stabilized within the composite polymer matrix. A parallel trend is also detected in the amide II region (1510–1550 cm^−1^), consistent with the enhanced ordering of the fibroin backbone. This SF conformational state and extensive protein crystallization may be attributed to the mechanical foaming process, likely acting as initiator for SF crystallization while the addition of β-TCP is expected to further stabilize the composite matrix. These effects are maintained in the presence of PCL [30,31]. A high degree of SF crystallinity is, among others, crucial in providing improved mechanical properties to the scaffolds.

### 3.2. Morphological Macroscopic Characterization and Interaction with Fluids and Dynamic Swelling

The morphological analysis was performed through stereoscopy and confirmed by scanning electron microscopy (SEM) for dry samples, or through environmental SEM (ESEM) for samples in wet conditions. The main purpose was the assessment of porosity and pore size, which are fundamental parameters for successful bone and periodontal regeneration, as they are closely related to cellular access to nutrients, oxygen exchange, bone tissue and vascular ingrowth, inflammation control, and metabolite removal. Moreover, pore size and interconnection influence scaffold permeability and regulate the rate of de novo tissue formation, while pore geometry is directly involved in osteogenesis [5,32,33].

**Figure 2:**
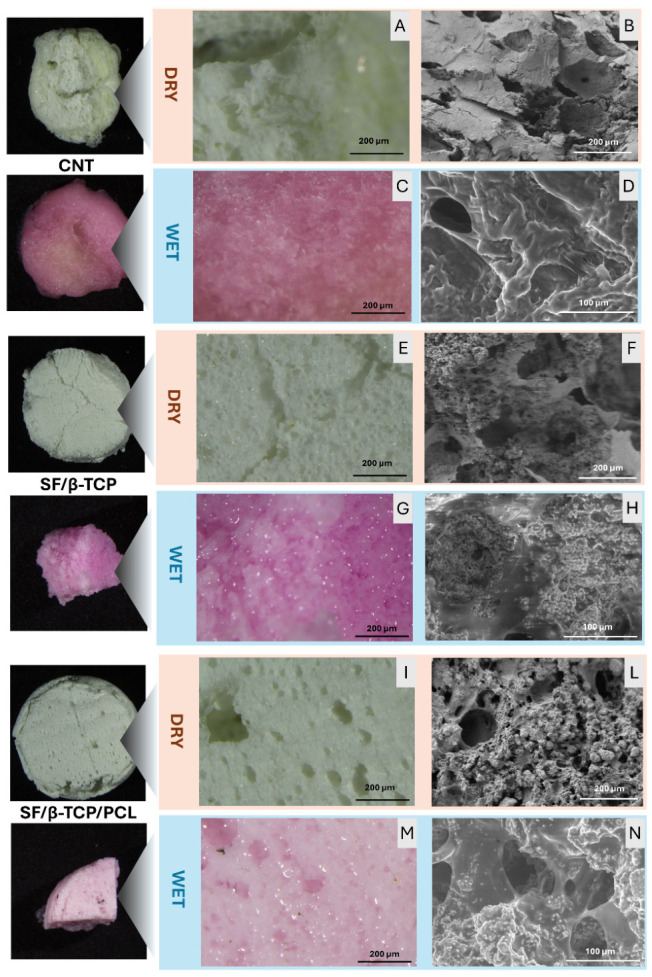
Overall morphological analysis of the samples under dry and wet conditions. Panels (**A**,**E**,**I**) display stereoscopic images of dry CNT, SF/β-TCP, and SF/β-TCP/PCL samples, respectively. Panels (**B**,**F**,**L**) show SEM micrographs of the same samples in dry state. Panels (**C**,**G**,**M**) present stereoscopic images of CNT, SF/β-TCP, and SF/β-TCP/PCL scaffolds after 1 week of soaking in DMEM at 37 °C, while the corresponding ESEM images acquired under wet conditions are shown in panels (**D**,**H**,**N**).

To mimic a biological application scenario, stereoscopic and ESEM images of the wet samples were acquired after 24 h and 1 week of soaking in HPLM-like medium and DMEM at 37 °C, respectively. Figure 2 shows the three samples of synthesized scaffolds under both dry and wet conditions, highlighting the differences in pore size and morphology. Great porosity and pore interconnection was observed in the SF/β-TCP/PCL samples, in both dry and wet conditions (Figure 2I,L,M,N), showing interconnected pores ranging from 20 to 200 µm and a high β-TCP content embedded within the scaffold’s matrix. This porosity was almost preserved in wet samples.

Dry SF/β-TCP samples showed good porosity and interconnectivity (Figure 2E,F) maintained. After the soaking step, the sample swelling led to a reconfiguration of pore size while maintaining morphology (Figure 2G,H). Finally, a marked swelling was observed in the CNT samples, resulting, from a macroscopical point of view, in a significant reduction in pore size and worsened morphology (Figure 2B,C). This behavior can be attributed to the amphipathic nature of SF, which causes substantial water uptake within the scaffold matrix [34,35]. This effect is reduced in the SF/β-TCP and SF/β-TCP/PCL samples. In particular, PCL incorporation may confer additional hydrophobicity to the matrix, thereby preventing excessive swelling and preserving pores after 1 week of soaking in DMEM [36,37].

The results highlight the importance of scaffold stability under physiological conditions, as supported by the measured bulk density values of 0.341, 0.439, and 0.476 g/cm^3^ for SF, SF/β-TCP, and SF/β-TCP/PCL scaffolds, respectively, which reflect the progressive incorporation of denser components while remaining within the range generally considered optimal for bone tissue regenerative applications [38].

We analyzed the swelling behavior of the samples using a standard gravimetric test, by measuring the water uptake within the scaffold matrix. Two sets of experiments were performed to determine the conventional swelling ratio (Sr) and total retained water (Rw) (Table 1), respectively. The analysis of Sr for the SF/β-TCP samples shows that the addition of β-TCP markedly affects the amount of water retained in the scaffold matrix, thereby influencing the final scaffold mass. Since β-TCP interacts weakly with water compared to pure SF matrix, a significant reduction in Sr was observed. This effect is even more pronounced in the SF/β-TCP/PCL samples, where a substantial decrease in the percentage of adsorbed water takes place. This behavior may be attributed to PCL incorporation into the matrix, which sterically shields SF interaction sites, thus minimizing the amount of exposed amphiphilic SFs available for water interaction.

Accordingly, Sr decreased to 18%. We further explored SF–solvent interactions by adapting the standard protocol: Rw was measured without removing excess water and the weights of wet scaffolds were compared before and after surface water removal (see Section 2 for details). This approach yielded the Rw parameter, which reflects water retained within material surfaces, pores, and cavities through surface tension and capillarity. The measured Rw values (Table 1) show an inverse trend compared to Sr, reflecting the higher porosity preserved in SF/β-TCP and SF/β-TCP/PCL scaffolds. These samples can still interact superficially with water, retaining only a minimal amount due to the exposed SF domains, while presumably allowing the exchange of nutrients and waste products at the surface. Notably, the SF/β-TCP/PCL samples exhibited an Rw of 48.1%, confirming the retention of substantial porosity under wet conditions.

Scaffold swelling is also crucial for the transport of nutrients and waste within matrices [39,40]. However, limiting excessive swelling is necessary to maintain equilibrium for homogeneous degradation, controlled release of loaded compounds, preservation of mechanical properties, and prevention of excessive pressure on the surrounding tissues [41,42,43,44]. Anti-swelling properties are typical of materials exhibiting an equilibrium water uptake ratio (or volume expansion rate) below 1.5. In recent years, anti-swelling hydrogels have shown great potential in various emerging applications, including tissue grafts, implantable artificial organs, tissue engineering, and regenerative medicine.These materials combine structural stability, adaptability, and cellular adhesiveness, overcoming several compatibility issues associated with traditional hydrogels when interfacing with dynamic or physiological environments [45]. An anti-swelling behavior in SF-based cryogenic materials has been recently reported by Niu et al., albeit in porous cryogels designed for cartilage defect repair [46].

In the present case, SF/β-TCP/PCL scaffolds initially exhibited partial anti-swelling properties, likely confined to the core of the matrices, while the surface retained the ability to interact with water. The synergy between these apparently contrasting features may be favorable for scaffold–environment interactions connected to swelling and to the preservation of morphological and mechanical stability observed during the initial days of exposure to the medium.

**Figure 3:**
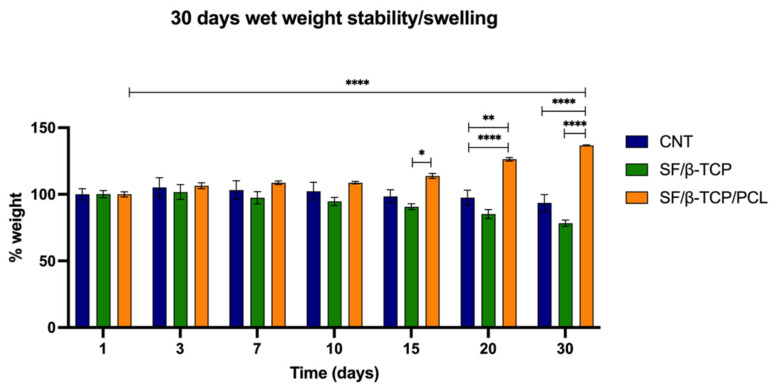
Analysis of wet weight stability/swelling monitoring performed over 30 days. The analysis was performed on CNT, SF/β-TCP and SF/β-TCP/PCL samples in PBS at 37 °C. Statistical significance was monitored by two-way analysis of variance (ANOVA). Data are presented as average ± SEM. SF/β-TCP/PCL day 1 vs. SF/β-TCP/PCL day 30, CNT day 20 vs. SF/β-TCP/PCL day 20, CNT day 30 vs. SF/β-TCP/PCL day 30, SF/β-TCP day 30 vs. SF/β-TCP/PCL day 30 **** *p* < 0.0001; SF/β-TCP day 15 vs. SF/β-TCP/PCL day 15 * *p* = 0.0372; CNT Day 20 vs. SF/β-TCP day 20 ** *p* = 0.0026.

To test this hypothesis, scaffolds’ behavior was monitored in PBS at 37 °C for 30 days (Figure 3) to mimic physiological conditions. Within this setup, the wet weight of the scaffolds was measured at different time points to assess swelling behaviors and weight fluctuations. After 30 days of exposure, the CNT samples showed negligible changes in weight, whereas SF/β-TCP samples exhibited a slight decrease in weight. In contrast, SF/β-TCP/PCL samples displayed a clear tendency toward weight gain.

Specifically, after 30 days, the SF/β-TCP/PCL scaffold exhibited a statistically significant change in weight compared to both the CNT and SF/β-TCP samples. In contrast, no meaningful weight variation was detected between CNT and SF/β-TCP, indicating that these materials had reached their maximum water uptake. After 30 days, the estimated Sr for SF/β-TCP/PCL samples was 74.2 ± 6.04%, representing a 411% increase compared to the 18% observed after 24 h (Table 1). Although the final Sr value remains relatively low, this behavior differs from the usual described for similar materials due to the hybrid swelling properties of SF/β-TCP/PCL [47,48]. Indeed, the medium infiltration into the SF/PCL matrix gradually triggers fluid adsorption and material swelling. This phenomenon could be further amplified by partial degradation of the external SF portion in the SF/β-TCP scaffolds, which tend to lose weight over time, likely as a consequence of degradation processes enhanced by their higher surface-to-volume ratio that remains nearly unaltered in CNT.

Specifically, SF degradation within the SF/β-TCP/PCL scaffolds facilitate a deeper infiltration of the medium into the SF/PCL structure. In this context, SF degradation weight loss is counterbalanced by enhanced water adsorption. It is worth noting that for this type of sample, achieving a high Sr is not feasible due to the high percentages of water insoluble β-TCP and PCL, both weakly interacting with water.

**Figure 4:**
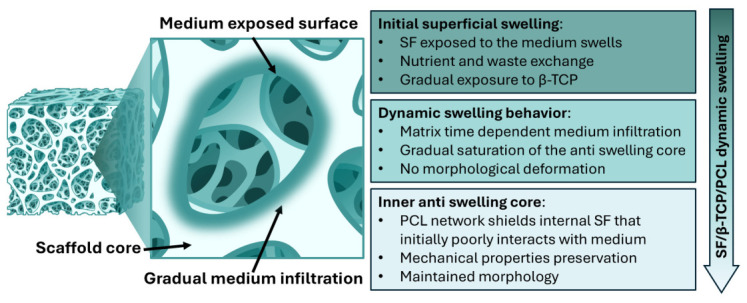
Schematic summary of SF/β-TCP/PCL samples’ dynamic swelling properties.

The observed dynamic swelling behavior may be relevant for tissue engineering applications, as it combines the retention of mechanical properties with a gradual material adaptation that could support initial cellular adhesion and scaffold colonization [49,50]. No substantial variations in overall scaffold size or deformation, which could presumably induce inflammatory responses after implantation, were detected. The reduced size fluctuations observed are likely attributable to the high amount of β-TCP loaded that poorly interacts with water, preserves the structural integrity, and prevents excessive swelling, together with the PCL network that overtime stabilizes the structure after prolonged exposition to water. Figure 4 provides a graphical summary of dynamic swelling and morpho-structural properties of the SF/β-TCP/PCL samples.

### 3.3. Assessment of Trabecular-Like Architecture Through µ-CT

**Figure 5:**
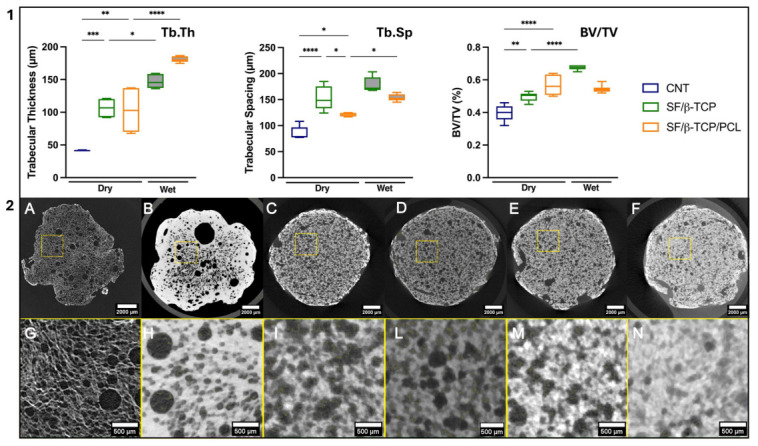
µ-CT evaluation of internal trabecular-like morphology of scaffolds. In panel (**1**), box-and-whisker plots based on descriptive and non-parametric analyses of Tb.Th, Tb.Sp, and BV/TV parameters, graphically presented in both wet and dry conditions (where applicable). Tb.Th: SF/β-TCP dry vs. SF/β-TCP dry wet * *p* = 0.0257; CNT dry vs. SF/β-TCP/PCL dry ** *p* = 0.0012; CNT dry vs. SF/β-TCP dry *** *p* = 0.0007; SF/β-TCP/PCL dry vs. SF/β-TCP/PCL wet **** *p* < 0.0001. Tb.Sp: CNT dry vs. SF/β-TCP/PCL dry * *p* = 0.0125; SF/β-TCP dry vs. SF/β-TCP/PCL dry * *p* = 0.0273; SF/β-TCP/PCL dry vs SF/β-TCP/PCL wet * *p* = 0.0255; CNT dry vs. SF/β-TCP **** *p* < 0.0001. BV/TV: CNT dry vs. SF/β-TCP ** *p* = 0.0036; CNT dry vs. SF/β-TCP/PCL dry and SF/β-TCP dry vs. SF/β-TCP wet **** *p* < 0.0001). In panel (**2**) the µ-CT cross-sectional images of the investigated scaffold groups: CNT, SF/β-TCP, and SF/β-TCP/PCL. SF/β-TCP and SF/β-TCP/PCL samples were evaluated under both the dry and wet conditions, whereas CNT samples were analyzed only in the dry state because, in the absence of an inorganic phase, the X-ray contrast between the SF polymer and water was too low to enable reliable phase discrimination in wet specimens. Wet samples were prepared by immersion in PBS at 37 °C for 24 h. Panels (**A**–**F**) report representative full cross-sectional views, whereas panels (**G**–**N**) report enlarged images of the yellow boxed regions shown in (**A**–**F**). The regions in (**G**–**N**), outlined in yellow, represent one representative volume of interest (VOI) out of the three VOIs selected for porosity analysis. Specifically, images (**A**,**G**) correspond to dry CNT, images (**B**,**H**) to wet CNT, images (**C**,**I**) correspond to dry SF/β-TCP, images (**D**,**L**) to wet SF/β-TCP, images (**E**,**M**) to dry SF/β-TCP/PCL, and images (**F**,**N**) to wet SF/β-TCP/PCL. Scale bars: 2000 µm in (**A**–**F**) and 500 µm in (**G**–**N**).

To get a comprehensive overview of the material’s properties, we also evaluated the internal trabecular-like morphology of the samples. The SF/β-TCP and SF/β-TCP/PCL samples were assessed under both dry and wet conditions whereas CNT samples were examined only in the dry state, since, in the absence of an inorganic phase, the X-ray contrast between the SF polymer and water in wet conditions was too low to reliably distinguish the two phases. Wet samples were obtained through the immersion in PBS at 37 °C for 24 h. The parameters of the samples evaluated from *µ-CT* analyses were: trabecular thickness (Tb.Th) (the thickness of the walls between the pores), trabecular separation (Tb.Sp) (the distance between the walls of the pores, i.e., the diameter of the pores), bone volume (BV) (the volume (measured in µm^3^) of the solid phase in the volume of interest (VOI)), total volume (TV) (the VOI measured in µm^3^). In Figure 5, Panel 1, box-and-whisker plots based on descriptive and non-parametric analyses of Tb.Th, Tb.Sp, and BV/TV parameters for the CNT, SF/β-TCP SF/β-TCP/PCL samples are shown; while in Panel 2 the respective cross-sectional images of the investigated scaffold groups are displayed.

The results of dry sample examination showed a clear structural separation for the variables of interest in the three groups. Tb.Th mean was markedly lower in CNT (40.7 µm) than in both SF/β-TCP (106 µm) and SF/β-TCP/PCL (103 µm), with significant overall differences and pairwise differences between CNT and each composite group, while the two composites were comparable. Tb.Sp mean was the highest in SF/β-TCP (153 µm), intermediate in CNT (140 µm), and the lowest in SF/β-TCP/PCL (119 µm); the overall group effect was significant, but the only significant pairwise difference was between SF/β-TCP and SF/β-TCP/PCL. BV/TV increased progressively from SF (0.396) to SF/β-TCP (0.498) and SF/β-TCP/PCL (0.567), indicating a denser structure in the composite scaffolds, with CNT being significantly lower than both other groups. Overall, SF/β-TCP and especially SF/β-TCP/PCL appear to be characterized by thicker trabeculae and higher volume fraction, while the CNT sample has a less massive architecture. This analysis conducted on dry samples reflects the overall higher amount of material loaded in the volume fraction analyzed (10% PCL) in SF/β-TCP/PCL composites, especially looking at the BV/TV parameter. The PCL loading also impacts on Tb.Th, indicating the composite incorporation and subsequent network formation into the trabecular area. We can also notice that the addition of PCL followed by the chloroform dipping step acted as functionally meaningful step, selectively reinforcing the load-bearing elements (thickening trabeculae) of the scaffold while maintaining porosity.

The heterogeneous yet rounded porosity that was maintained may positively influence bone regeneration, as it is now well established that bone is more heterogeneous and complex than previously assumed [51]. Furthermore, the formation and preservation of pores’ concave geometry represents a key advantage, since bone tissue regeneration preferentially occurs on concave surfaces [52].

Under wet conditions, both groups maintained the same overall pattern, but SF/β-TCP/PCL showed a clearly more massive architecture than SF/β-TCP. In particular, Tb.Th mean was significantly higher in SF/β-TCP/PCL (186 µm) than in SF/β-TCP (147 µm), while Tb.Sp mean was significantly lower in SF/β-TCP/PCL (169 µm) than in SF/β-TCP (191 µm), indicating a denser and more compact trabecular arrangement. Consistently, BV/TV was markedly higher in SF/β-TCP/PCL (0.674) than in SF/β-TCP (0.543), confirming the greater volume fraction of solid structure in the PCL-containing group.

Based on the descriptive values, moving from dry to wet state, both composite groups showed a shift toward a more massive structure, with a higher Tb.Th mean and higher BV/TV. In SF/β-TCP, Tb.Th mean increased from 106 to 147 µm and BV/TV from 0.498 to 0.543, while Tb.Sp mean also increased from 153 to 191 µm. In SF/β-TCP/PCL, the same tendency was even more evident: Tb.Th mean increased from 103 to 186 µm, BV/TV from 0.567 to 0.674, and Tb.Sp mean from 119 to 169 µm. Overall, the wet condition appears to be associated with thicker trabeculae and a higher volume fraction in both materials; at the same time, SF/β-TCP/PCL preserved the highest Tb.Th, and BV/TV in both conditions SF/β-TCP/PCL is characterized by significantly thicker trabeculae. This suggest that, despite being more hydrophobic, with an inner core initially preserved from the interaction with water, the superficial layers of the trabeculae start to swell after 24 h, establishing a strong interaction with water and leading to a higher bone volume fraction while preserving an increase in Tb.Sp mean (169 µm).

In overall dry vs. wet comparisons, both materials showed a statistically significant shift in the same general direction: in the wet state, both SF/β-TCP and SF/β-TCP/PCL had higher Tb.Th mean, higher Tb.Sp mean, and higher BV/TV. In SF/β-TCP, Tb.Th mean increased from 106.5 to 147.1 µm (*p* < 0.001), Tb.Sp mean from 152.5 to 191.2 µm (*p* = 0.038), and BV/TV from 0.498 to 0.543 (*p* = 0.009). The same pattern was even more pronounced in SF/β-TCP/PCL. Tb.Th mean rose from 103.1 to 186.2 µm (*p* < 0.001), (*p* = 0.012), and BV/TV from 0.567 to 0.674 (*p* = 0.002). Overall, wetting appears to produce a structure that is thicker but still able to preserve connectivity in both groups, with the effect being markedly stronger in SF/β-TCP/PCL.

An interesting observation concerns the relationship between Tb.Sp and Tb.Th in SF/β-TCP/PCL scaffolds. Specifically, we observed a 41% increase in Tb.Sp and an 80% increase in Tb.Th after 24 h of soaking. This suggests that SF/β-TCP/PCL scaffolds can hypothetically preserve a satisfactory pore morphology and a higher Rw value due to a more rigid trabecular-like structure provided by the PCL network. This behavior may arise from the dynamic swelling process and an initially less water-interactive inner portion of the scaffold, as indicated by the early limited water uptake and swelling by SF/β-TCP/PCL composites and relatively low Sr value after 24 h of soaking (see Table 1). In Figure 5, it is also worth noting the limited presence of a dense surface layer (surface sealing) in all conditions. The images were acquired without any additional surface treatment, indicating that cryogenic foaming significantly reduces this common issue observed in other fabrication methods [13,17].

### 3.4. Rheological Analysis Through Uniaxial Compression Tests of Scaffolds

The mechanical properties of composite scaffolds were investigated under dry and wet conditions. CNT and SF/β-TCP scaffolds exhibited comparable mechanical behavior, whereas the SF/β-TCP/PCL scaffold showed a significantly higher compressive modulus in both environments. The elastic modulus of CNT scaffolds is consistent with values reported in a previous study [21].

The addition of 10% PCL enhanced the rigidity of the scaffold, regardless of the hydration state. This formulation resulted in stiffer samples than those developed for bone tissue engineering and, considering the resistance to degradation discussed above, suggests a possible retention of the mechanical properties, even for a prolonged regenerative process [53,54].

**Figure 6:**
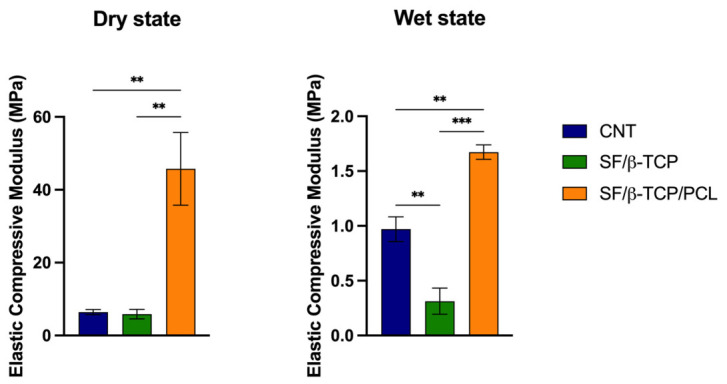
Elastic compressive modulus evaluated in dry and wet conditions (at T0 and after 24 h of immersion in PBS) for CNT, SF/β-TCP and SF/β-TCP/PCL. Data are presented as average ± SEM. Dry state: CNT vs. SF/β-TCP/PCL ** *p* = 0.0073, SF/β-TCP vs. SF/β-TCP/PCL ** *p* = 0.0069. Wet state: CNT vs. SF/β-TCP ** *p* = 0.0091, CNT vs. SF/β-TCP /PCL ** *p* = 0.0065, SF/β-TCP vs. SF/β-TCP/PCL *** *p* = 0.0002.

Nevertheless, after 24 h of soaking in PBS, all scaffolds exhibited an approximately 10-fold reduction in mechanical resistance, with the SF/β-TCP/PCL scaffold still maintaining the highest performance (Figure 6). Uniaxial compression tests of dry (Figure S1) and wet scaffolds (Figure S2) are reported in the Supplementary Materials. This decrease in stiffness is likely related to the amphipathic nature of these constructs [53]. Although the results indicate an improvement, particularly for the SF/β-TCP/PCL scaffolds, their mechanical strength still requires further improvements, as the elastic modulus does not yet fully match that of the native cancellous bone, (≈90–400 MPa) [55]. Consequently, in the actual scenario, the proposed scaffolds may be suitable as fillers for subcritical defects or in combination with mechanically supportive frameworks [56].

Comparing the present approach to a gas foaming method using same components, our technique allowed the incorporation of more than 200% of ceramic material using SF as main component of the scaffold instead of PCL while maintaining comparable mechanical properties [13].

### 3.5. 3D Culture and Cell Morphology on Scaffolds

**Figure 7:**
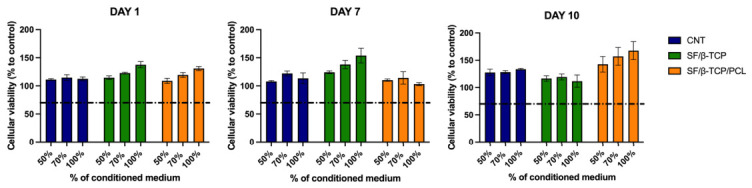
Indirect contact cytotoxicity assays of L929-murine fibroblasts according to ISO 10933-5 guidelines on different SF-based scaffolds. The dotted line represents the biocompatibility threshold set at 70%. Data are presented as average ± SEM.

A preliminary exploration of scaffold’s biocompatibility was performed using an indirect cytotoxicity assay with varying concentrations of the conditioned medium (Figure 7). The results indicated no significant negative effect on cellular viability, as all tested time points (1, 7, and 10 days of immersion) demonstrated cellular viability levels well above the 70% threshold, which is the minimum requirement for biocompatibility. Cell viability consistently exceeded this limit, indicating that the material does not exhibit cytotoxic effects. These findings confirmed the good compatibility of the scaffold components (i.e., SF, β-TCP, and PCL), as well as the absence of residual toxic compounds from the production process, suggesting the suitability of the proposed scaffold for biomedical applications.

The results of the indirect cytotoxicity assay were further corroborated by evaluating cell proliferation on the composite scaffolds. For this reason, MG-63 osteoblast-like cells were seeded onto the scaffold surfaces, and their early adhesion and subsequent growth were monitored over time up to 1 week of culture. To assess cellular activity, MTT assays were performed, allowing for a quantitative evaluation of cell proliferation. These analyses further support scaffold biocompatibility, demonstrating the ability of the cells to adhere to the material, proliferate, and maintain metabolic activity under the tested conditions.

**Figure 8:**
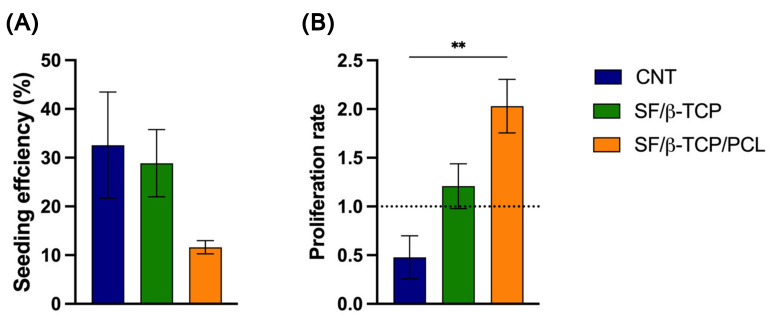
Biocompatibility of the three SF-based composite scaffolds in terms of MG-63 adhesion and proliferation. (**A**) The seeding efficiency after 24 h was compared to that of a cell line cultured in standard 2D well plates as a reference. (**B**) The proliferation rate after 7 days of culture was compared and normalized to day 1 (represented by the grid line). Data are presented as average ± SEM. CNT vs. SF/β-TCP/PCL ** *p* = 0.0096.

The results of the proliferation assays are consistent with the cytotoxicity testing, further supporting the non-toxic nature and initial favorable biological interaction of the composite scaffolds (Figure 8).

Cell adhesion was initially evaluated 24 h after seeding on the proposed scaffold compositions, revealing measurable differences. As a control, 2D cells cultured in a multiwell plate were used for comparison. Both the CNT and SF/β-TCP scaffolds showed comparable adhesion behavior, whereas the SF/β-TCP/PCL scaffolds displayed a slightly reduced seeding efficiency. This decreased efficiency is likely attributable to the hydrophobic nature of PCL, which may affect the initial cell attachment.

Cell proliferation rate was re-evaluated after 1 week of culture, with the results normalized to day 1 to enable the comparison of differences in cell behavior across the three scaffold compositions over time. Despite higher initial cell adhesion, the CNT scaffold exhibited a decrease in cell number after 1 week. In contrast, the SF/β-TCP scaffold showed a slight increase in cell number over the same period. Notably, the SF/β-TCP/PCL scaffold outperformed the other formulations, exhibiting robust cell proliferation and supporting sustained, long-term cell growth on the scaffold surface. This behavior suggests that the inclusion of PCL may contribute to improved cell expansion over time under the tested conditions. Moreover, the presence of β-TCP into the fibroin matrix participates to the observed favorable biological response by enhancing scaffold surface characteristics. Indeed, nanostructured surfaces can increase surface/volume ratio and overall surface available for bonding with water, thereby increasing protein absorption and promoting cellular adhesion and proliferation, while also providing a larger effective surface area for osteoblast attachment [57,58,59,60].

**Figure 9:**
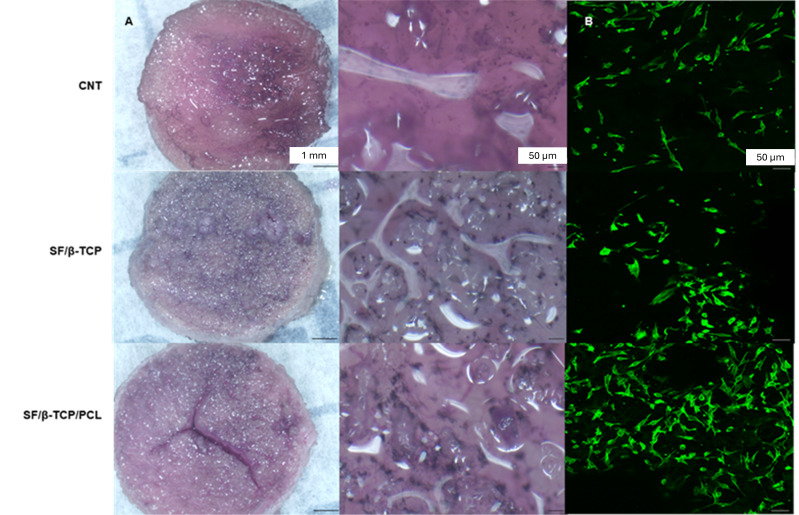
Cell distribution and adhesion: (**A**) Stereoscope images demonstrated a good cell colonization of the scaffold surface after a week of culture. (**B**) Confocal fluorescent images confirmed a cell shape compatible with good adhesion of the cells with the fibroin samples.

Stereoscope images (Figure 9) acquired after the MTT assay, showed the distribution of the formazan crystals on different surfaces, indicating metabolically active cells homogeneously distributed around the surfaces. In the confocal images, the phalloidin-labeled cytoskeleton showed extended cell structures, which is an indication of good cell adhesion. Confocal fluorescent images further confirmed cell morphology, which appeared well-suited for optimal adhesion to the fibroin-based scaffold samples. The images revealed a characteristic cell shape that is indicative of good cell attachment and spreading, reinforcing the positive results observed in the adhesion assays. These visual observations align with the favorable cell behavior seen on the fibroin scaffolds, confirming that the material supports not only cell attachment but also proper cellular function and growth at early time points.

The proliferation results can be explained by the behavior of the hydrated scaffold. A reduction in pore size limits the exchange of metabolites for cells growing on the scaffold, which may limit nutrient exchange and potentially affect cell viability, as observed for CNT. In contrast, the structure stability of SF/β-TCP/PCL ensures adequate media exchange, supporting cell viability and enabling proliferation.

Beyond these morphological features, the observed behavior may also be influenced by the ability of the osteoblast to sense substrate stiffness through mechanotrasduction mechanisms, further influencing cell proliferation and fate [61]. Our results are consistent with Sun et al.’s findings, who reported a higher proliferation rate of BMMSCs on stiffer substrates (62–68 kPa), whereas a reduction of 40% was reported on softer substrates (13–16 kPa) [62]. Moreover, increased matrix stiffness has been associated with enhanced osteogenic differentiation [63]. In in vitro models, this effect has been attributed to stiffness-dependent mechanotrasduction mechanisms involving TAZ, including the Polycystin–TAZ complex, the YAP/TAZ signaling axis, and MIF-mediated AKT/YAP/Runx2 pathways, which collectively regulate osteogenic commitment [64]. Nevertheless, it is important to highlight that the presented biological tests are preliminary and limited to short-term in vitro evaluation of biocompatibility response as they were conducted primarily to assess the influence of the production methodology on material’s biocompatibility. While these initial findings provide useful insights related to cellular adhesion and proliferation, further studies, including long-term osteogenic differentiation assays, advanced 3D culture models, and in vivo investigations are required to fully assess the regenerative potential of the proposed scaffolds.

## 4. Conclusions

In this study, composite scaffolds for bone and periodontal regeneration were synthetized through cryogenic mechanical foaming, a simple, innovative and cost-effective fabrication method that enables the production of highly porous structures with a large surface and a high surface/volume ratio. Compared to other synthesis methodologies, this approach allows the formation of rounded pore morphologies, resulting from the bubble shape generated during mechanical foaming and preserved through the cryogenic step. Also, the phenomenon of surface sealing was drastically reduced.

Three scaffold formulations were successfully characterized: neat SF (CNT), SF with 30% *w*/*v* β-TCP (SF/β-TCP), and SF with 30% *w*/*v* β-TCP and 10% *w*/*v* PCL (SF/β-TCP/PCL). The synthesis method enabled the integration of ceramic components, which are essential for enhancing the osteogenic potential of scaffolds intended for bone regenerative applications, reaching the incorporation of up to 30% *w*/*v* β-TCP and 10% *w*/*v* PCL.

The addition of β-TCP improved surface topography by introducing a micro-rough morphology that promoted cell attachment. The addition of PCL further optimized the rheological, mechanical, and biological properties of the grafts, and especially in SF/β-TCP/PCL samples, we observed a dynamic swelling behavior over time. PCL absorption into the trabecular matrix was achieved through a step that involved soaking in chloroform, allowing both the dissolution and the homogeneous integration of PCL into the scaffold matrix, thereby markedly stabilizing the structure and significantly enhancing the mechanical performances under both dry and hydrated conditions.

These structural and mechanical improvements were translated through the biological aspect, as stiffer substrates ameliorated the adhesion and proliferation of osteoblasts up to 1 week of culture. Nevertheless, further optimizations of mechanical properties are still required to fully meet the physiological demands of the alveolar bone.

This fabrication method yields a material whose structure mimics bone tissue, offering a competitive and original alternative to recently reported composites, which typically require more complex synthesis procedures.

In conclusion, owing to their high porosity, bioactivity, structural stability, and adequate biological properties, the proposed scaffolds might be a highly promising candidate for subsequent extensive in vitro validation related to osteoblastic differentiation and consequent in vivo testing as bone grafting material.

## Figures and Tables

**Table 1:** Sr and Rw values calculated for CNT, SF/β-TCP, and SF/β-TCP/PCL samples after 1 day of soaking in ultrapure water. Data are presented as average ± SEM.

% Swelling Ratio (Sr)			% Total Retained Water (R_W_)		
CNT	260.2	±21.7	CNT	18.8	±6.7
SF/β-TCP	113.7	±4.7	SF/β-TCP	36.0	±5.6
SF/β-TCP/PCL	18.0	±0.4	SF/β-TCP/PCL	48.1	±2

## Data Availability

Data related to the present project are available upon reasonable request to the corresponding authors.

## Supplementary Materials

The following supporting information can be downloaded at: https://www.mdpi.com/article/10.3390/jfb17050230/s1.

## Abbreviations

The following abbreviations are used in this manuscript:

SF	silk fibroin
β-TCP	β-tricalcium phosphate
PCL	poly-ε-caprolactone
CNT	control samples composed solely of pure SF
BTE	bone tissue engineering
ECM	extracellular matrix
HA	hydroxyapatite
ATR-FTIR	Attenuated Total Reflectance-Fourier Transform Infrared
SEM	scanning electron microscopy
eSEM	environmental scanning electron microscopy
µ-CT	X-ray microcomputed tomography
S_r_	swelling ratio
R_w_	total retained water
LiBr	lithium bromide
PLA	polylactic acid
FBS	fetal bovine serum
MTT	tetrazolium salts test
DMSO	dimethyl-sulfoxide
PBS	phosphate-buffered saline
SEM	standard error of the mean
ANOVA	analysis of variance
