# Supplementary material for: Cryogenic Foaming of Silk Fibroin Composite for Scaffolds in Bone and Periodontal Regeneration
Source: J Funct Biomater. 2026 May 6;17(5):230. doi: 10.3390/jfb17050230 (PMC13207308; doi:10.3390/jfb17050230)
Supplement: Supplementary file 1 [file jfb-17-00230-s001.zip › jfb-4261954-supplementary.pdf]

Journal of
Functional Biomaterials

MDPI

SUPPLEMENTARY SECTION

Article

Cryogenic Foaming of Silk Fibroin Composite for Scaffolds in Bone and Periodontal Regeneration

Giuseppe De Giorgio[1], Barbara Medagli[2], Biagio Matera[3*], Katia Rupel[2], Giuseppe Tarabella[1], Gianluca Turco[2], Maddalena Manfredi[3], Benedetta Ghezzi1[1,3*], Pasquale D'Angelo[1]

1 Institute of Materials for Electronics and Magnetism (IMEM-CNR), Parco Area delle Scienze 37A, 43124 Parma, Italy.
2 Clinical Department of Medical, Surgical and Health Sciences, University of Trieste, 34100, Trieste, Italy
3 Centre of Dental Medicine, Department of Medicine and Surgery, University of Parma, Via Gramsci 14/A, 43126 Parma, Italy.

* Corresponding authors:
benedetta.ghezzi@unipr.it; Tel 0039 0521 906742; Centre of Dental Medicine, Department of Medicine and Surgery, University of Parma, Via Gramsci 14/A, 43126 Parma, Italy

biagio.matera@unipr.it; Tel 0039 0521 906741; Centre of Dental Medicine, Department of Medicine and Surgery, University of Parma, Via Gramsci 14/A, 43126 Parma, Italy

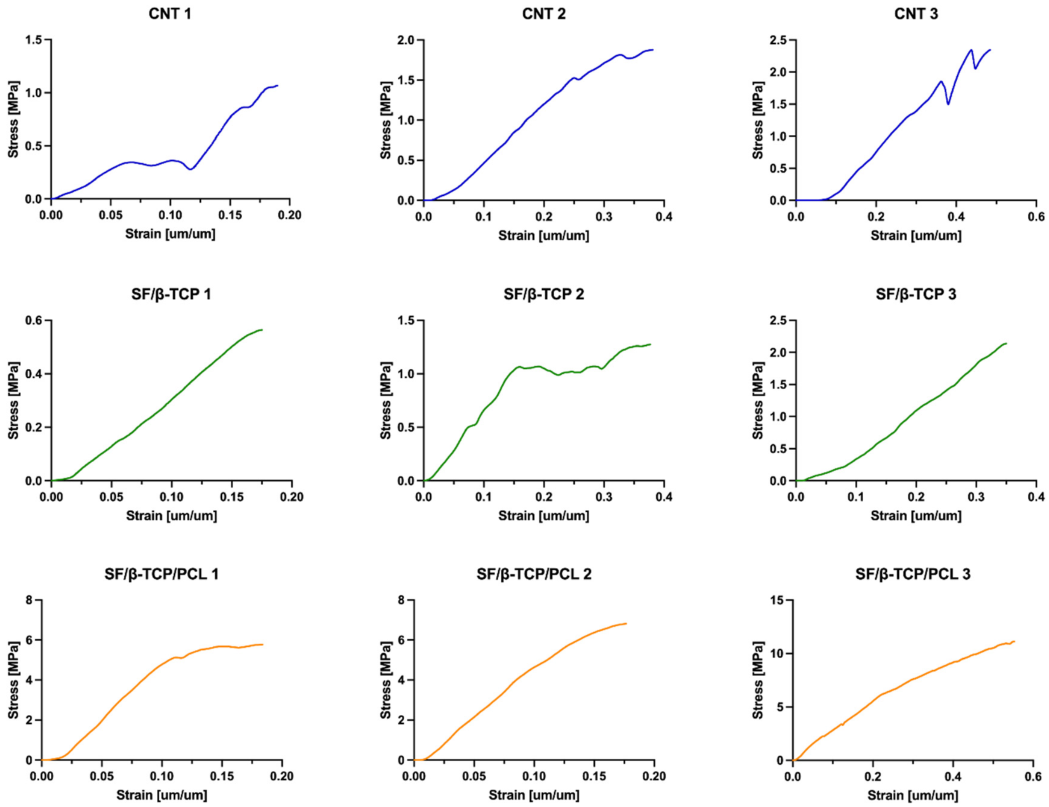

Figure S1. Uniaxial compression tests of dry scaffolds: The Stress/Strain graphs were plotted normalizing the recorded data on the surface of the pressor and the initial length of the scaffold.

Journal of
Functional Biomaterials

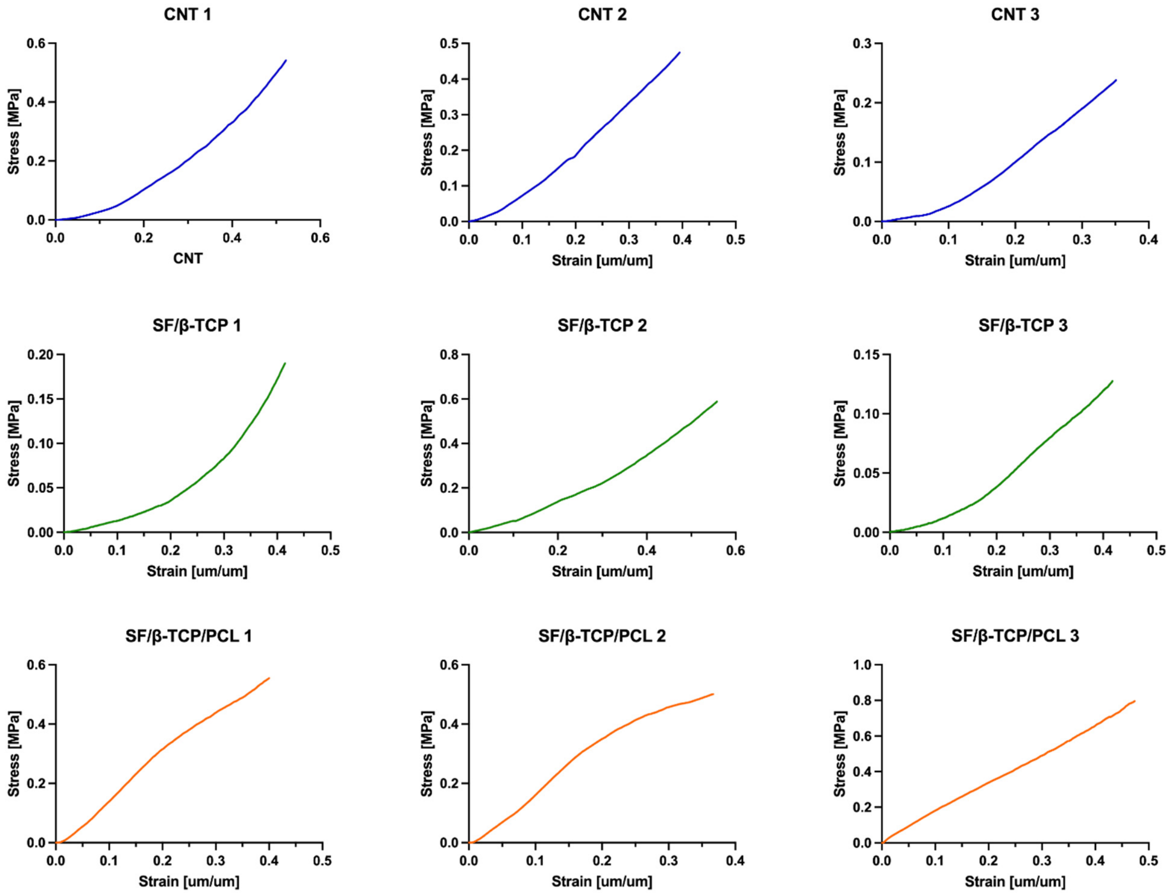

Figure S2. Uniaxial compression tests of wet scaffolds: The Stress/Strain graphs were plotted normalizing the recorded data on the surface of the pressor and the initial length of the scaffold.
